# 10-{4-[(2-Hy­droxy­benzyl­idene)amino]­phen­yl}-5,5-di­fluoro-1,3,7,9-tetra­methyl-5*H*-di­pyrrolo­[1,2-*c*:2′,1′-*f*][1,3,2]di­aza­borinin-4-ium-5-uide

**DOI:** 10.1107/S1600536813015523

**Published:** 2013-06-12

**Authors:** Zhensheng Li

**Affiliations:** a Key Laboraory of Photochemical Conversion and Optoelectronic Materials, Technical Institute of Physics and Chemistry, Chinese Academy of Sciences, Zhongguancun Donglu 29, 100190 Beijing, People’s Republic of China

## Abstract

The title compound, C_26_H_24_BF_2_N_3_O, comprises a boron–dipyrromethene (BODIPY) framework and a phenolic Schiff base substituent group. The BODIPY unit is close to planar [maximum deviation from the least-squares plane = 0.040 (3) Å], and forms a dihedral angle of 80.38 (13)° with the *meso*-substituent phenyl ring and an angle of 56.57 (13)° with the phenolic ring in the extended substituent chain. An intra­molecular O—H⋯N hydrogen bond is formed between the phenolic hydroxyl group and the Schiff base N-atom. The crystal studied was a non-merohedral twin with a BASF factor of 0.447 (3) for the two components.

## Related literature

For the photophysical properties of BODIPY dyes, see: Loudet & Burgess (2007 ▶); Boens *et al.* (2012 ▶). For the use of related compounds for fluorescence analysis, see: Fan *et al.* (2012 ▶); Li *et al.* (2012 ▶). For the preparation of the BODIPY precursor, see: Lu *et al.* (2009 ▶).
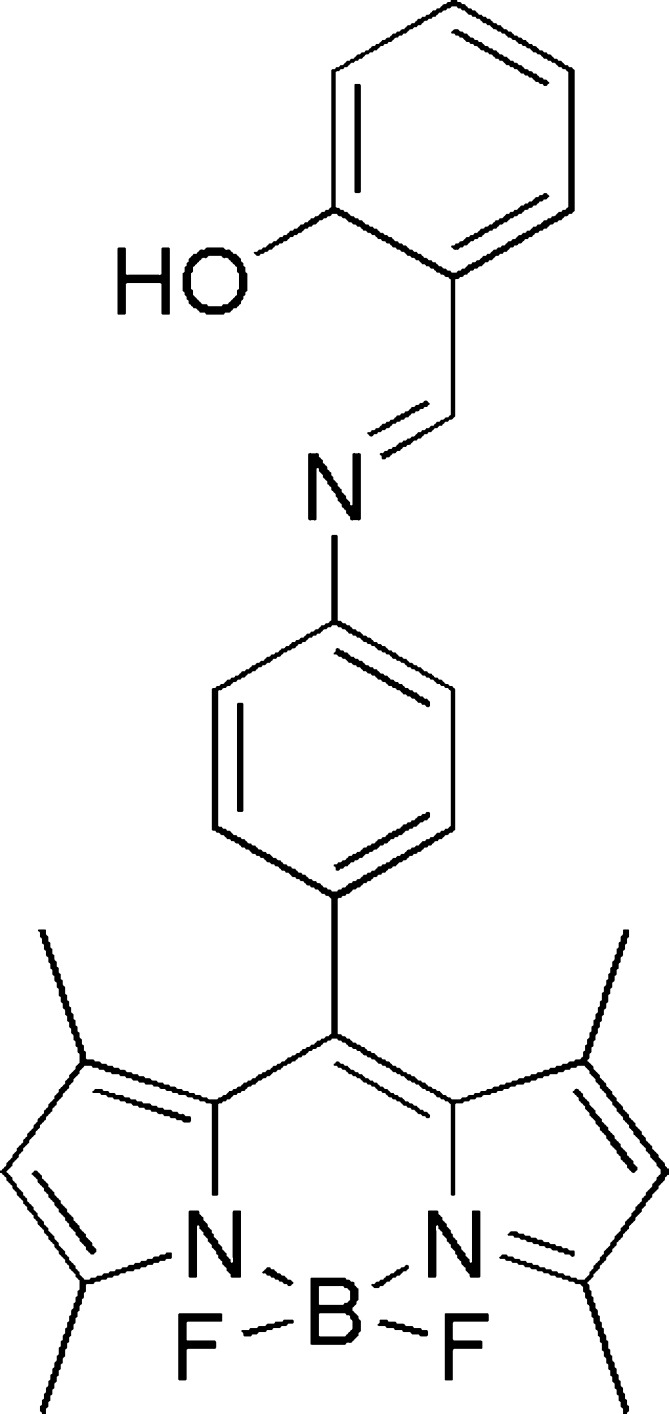


## Experimental

### 

#### Crystal data

C_26_H_24_BF_2_N_3_O
*M* *_r_* = 443.29
Triclinic, 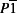

*a* = 8.8920 (15) Å
*b* = 10.7480 (17) Å
*c* = 12.9230 (18) Å
α = 110.258 (9)°
β = 90.952 (6)°
γ = 108.408 (7)°
*V* = 1088.3 (3) Å^3^
*Z* = 2
Mo *K*α radiation
μ = 0.10 mm^−1^
*T* = 113 K
0.24 × 0.20 × 0.18 mm

#### Data collection

Rigaku Saturn724 CCD-detector diffractometer
Absorption correction: multi-scan (*ABSCOR*; Higashi, 1995 ▶) *T*_min_ = 0.978, *T*_max_ = 0.983
12167 measured reflections
5171 independent reflections
2822 reflections with *I* > 2σ(*I*)
*R*_int_ = 0.054

#### Refinement

*R*[*F* ^2^ > 2σ(*F* ^2^)] = 0.071
*wR*(*F* ^2^) = 0.217
*S* = 1.09
5171 reflections
304 parameters
H-atom parameters constrained
Δρ_max_ = 0.77 e Å^−3^
Δρ_min_ = −0.44 e Å^−3^

### 

Data collection: *CrystalClear-SM Expert* (Rigaku, 2009 ▶); cell refinement: *CrystalClear-SM Expert*; data reduction: *CrystalClear-SM Expert* (Rigaku, 2009 ▶); program(s) used to solve structure: *SHELXS97* (Sheldrick, 2008 ▶); program(s) used to refine structure: *SHELXL97* (Sheldrick, 2008 ▶); molecular graphics: *PLATON* (Spek, 2009 ▶); software used to prepare material for publication: *CrystalStructure*.

## Supplementary Material

Crystal structure: contains datablock(s) I, global. DOI: 10.1107/S1600536813015523/zs2263sup1.cif

Structure factors: contains datablock(s) I. DOI: 10.1107/S1600536813015523/zs2263Isup2.hkl

Additional supplementary materials:  crystallographic information; 3D view; checkCIF report

## Figures and Tables

**Table 1:** Hydrogen-bond geometry (Å, °)

*D*—H⋯*A*	*D*—H	H⋯*A*	*D*⋯*A*	*D*—H⋯*A*
O1—H1⋯N3	0.84	1.89	2.618 (4)	145

**Fig. 1.**
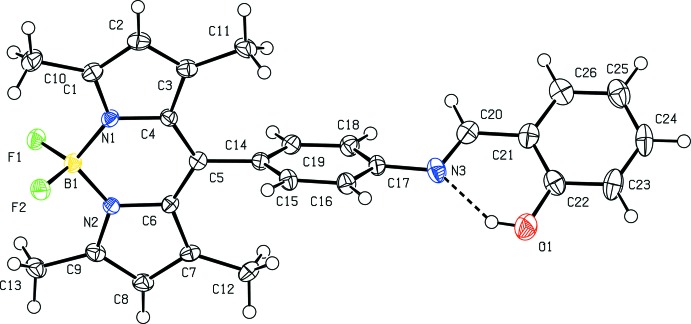
Molecular conformation and atom numbering scheme (PLATON, Spek, 2009) of the title compound, with displacement ellipsoids drawn at 50% probability level and H atoms with arbitrary radius. The intramolecular hydrogen bond is shwn as a dashed line.

**Fig. 2.**
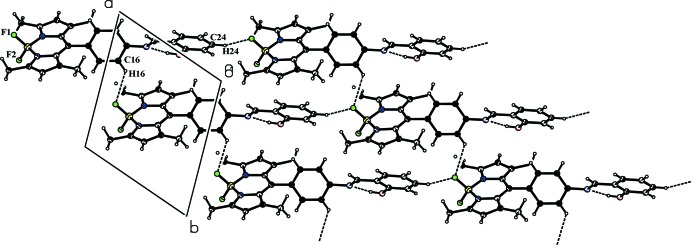
Tig. 2. The two-dimensional network structure extending along [110], formed by weak nonclassical intermolecular C–H···F hydrogen-bonding interactions.

## supplementary crystallographic information

### Comment

Among various fluorescent compounds, boron complexes, especially boron-dipyrromethene (BODIPY), show many photophysical advantages over other dyes, such as high absorption coefficients and fluorescence quantum yields, narrow emission spectra, excellent stability towards light and chemicals, enabling their applications in light harvesting, biological imaging and fluorescent probes (Boens *et al.*, 2012). As part of our ongoing studies of fluorescent probes for metal ions, we report herein the crystal structure of the title compound, 10-(4-((2-hydroxybenzylidene)amino)phenyl)-5,5- difluoro-1,3,7,9-tetramethyl-5*H*-dipyrrolo[1,2-c:2',1'-f][1,3,2] diazaborinin-4-ium-5-uide, C_26_H_24_BF_2_N_3_O.

The title compound comprises essentially a C_9_BN_2_ (BODIPY) parent component and a phenolic Schiff base substituent group (Fig. 1). The bond lengths and angles are within normal ranges [B—F, 1.397 (4), 1.402 (4) Å and B—N, 1.528 (4), 1.541 (4) Å]. The BODIPY core is close to planar, with a maximum deviation from the least-squares plane of 0.040 (3) Å. The *meso*-substituted benzene ring defined by atoms C14–C19 is essentially perpendicular to this plane, forming a dihedral angle of 80.38 (13) ° with it. The phenolic ring in the extended substituent chain, defined by atoms C21–C26, forms a dihedral angle of 56.57 (13)° with the BODIPY plane. A moderately strong intramolecular O—H···N hydrogen bond is formed between the phenolic hydroxyl group and the Schiff base N-atom (Table 1). Weak non-classical intermolecular aromatic C—H···F hydrogen bonds extend the molecules into two-dimensional network structure lying parallel to [110] (Fig. 2).

### Experimental

The BODIPY precursor 10-(4-Aminophenyl)-5,5-difluoro-1,3,7,9-tetramethyl-5*H*-dipyrrolo [1,2 - c:2,,1,-f][1,3,2]diaza-borinin-4-ium-5-uide was synthesized according to the literature procedure (Lu *et al.*, 2009). To a mixture of this compound (100 mg, 0.30 mmol) and 2-hydroxybenzaldehyde (50 mg, 0.40 mmol) in EtOH, one drop of trifluoroacetic acid was added. The reaction solution was heated at 353 K for 4 h, and the red precipitate was filtered and washed with ethanol to obtain the title compound. Yield: 80%. Red crystals. ^1^H NMR (400 MHz, CDCl_3_) δ (ppm.): 13.06 (s, 1H), 8.72 (s, 1H), 7.43 (dd, *J* = 12.4, 4.5 Hz, 4H), 7.36 (d, *J* = 8.0 Hz, 2H), 7.06 (d, *J* = 8.2 Hz, 1H), 6.98 (t, *J* = 7.5 Hz, 1H), 6.01 (s, 2H), 2.56 (d, *J* = 9.4 Hz, 6H), 1.46 (s, 6H). MALDI-TOF: m/*z* = 443.1 [*M*]^+^, 424.0 [M—F]^+^. Red single crystals suitable for X-ray analysis were obtained by dissolving the compound (0.10 g) in a hexane/dichloromethane (15 ml, *v*/*v*, 1/1) mixture and slowly evaporating the solvent at room temperature for a period of about one week.

### Refinement

Twinning was detected by *TwinRotMat* in *PLATON* (Spek, 2009) with a BASF factor of 0.45. A HKLF5 format reflection file was generated for the refinement, giving a final BASF factor of 0.447 (3). Hydrogen atoms were treated as riding with C—H = 0.95 Å for aryl-CH, C—H = 0.98 Å for CH_3_ groups and O—H = 0.84 Å for the hydroxy group. Isotropic displacement parameters for hydrogen atoms were constrained to *U*_iso_(H)= 1.2*U*_eq_(C) for aryl H-atoms and *U*_iso_(H) = 1.5*U*_eq_(C,O) for methyl and hydroxy groups.

### Crystal data

**Table:**

C_26_H_24_BF_2_N_3_O	*Z* = 2
*M**_r_* = 443.29	*F*(000) = 464
Triclinic, *P*1	*D*_x_ = 1.353 Mg m^−^^3^
Hall symbol: -P 1	Mo *K*α radiation, λ = 0.71075 Å
*a* = 8.8920 (15) Å	Cell parameters from 3734 reflections
*b* = 10.7480 (17) Å	θ = 1.7–27.9°
*c* = 12.9230 (18) Å	µ = 0.10 mm^−^^1^
α = 110.258 (9)°	*T* = 113 K
β = 90.952 (6)°	Prism, colorless
γ = 108.408 (7)°	0.24 × 0.20 × 0.18 mm
*V* = 1088.3 (3) Å^3^	

### Data collection

**Table:**

Rigaku Saturn724 CCD-detector diffractometer	5171 independent reflections
Radiation source: rotating anode	2822 reflections with *I* > 2σ(*I*)
Multilayer monochromator	*R*_int_ = 0.054
Detector resolution: 14.222 pixels mm^-1^	θ_max_ = 28.0°, θ_min_ = 1.7°
ω scans	*h* = −11→11
Absorption correction: multi-scan (*ABSCOR*; Higashi, 1995)	*k* = −14→14
*T*_min_ = 0.978, *T*_max_ = 0.983	*l* = −16→16
12167 measured reflections	

### Refinement

**Table:**

Refinement on *F*^2^	Primary atom site location: structure-invariant direct methods
Least-squares matrix: full	Secondary atom site location: difference Fourier map
*R*[*F*^2^ > 2σ(*F*^2^)] = 0.071	Hydrogen site location: inferred from neighbouring sites
*wR*(*F*^2^) = 0.217	H-atom parameters constrained
*S* = 1.09	*w* = 1/[σ^2^(*F*_o_^2^) + (0.1035*P*)^2^ + 0.5082*P*] where *P* = (*F*_o_^2^ + 2*F*_c_^2^)/3
5171 reflections	(Δ/σ)_max_ < 0.001
304 parameters	Δρ_max_ = 0.77 e Å^−^^3^
0 restraints	Δρ_min_ = −0.44 e Å^−^^3^

### Special details

**Table:**

Geometry. All e.s.d.'s (except the e.s.d. in the dihedral angle between two l.s. planes) are estimated using the full covariance matrix. The cell e.s.d.'s are taken into account individually in the estimation of e.s.d.'s in distances, angles and torsion angles; correlations between e.s.d.'s in cell parameters are only used when they are defined by crystal symmetry. An approximate (isotropic) treatment of cell e.s.d.'s is used for estimating e.s.d.'s involving l.s. planes.
Refinement. Refinement of *F*^2^ against ALL reflections. The weighted *R*-factor *wR* and goodness of fit *S* are based on *F*^2^, conventional *R*-factors *R* are based on *F*, with *F* set to zero for negative *F*^2^. The threshold expression of *F*^2^ > σ(*F*^2^) is used only for calculating *R*-factors(gt) *etc*. and is not relevant to the choice of reflections for refinement. *R*-factors based on *F*^2^ are statistically about twice as large as those based on *F*, and *R*- factors based on ALL data will be even larger.

### Fractional atomic coordinates and isotropic or equivalent isotropic displacement parameters (Å^2^)

**Table:**

	*x*	*y*	*z*	*U*_iso_*/*U*_eq_	
F1	0.8363 (2)	0.60168 (18)	0.29484 (15)	0.0237 (4)	
F2	0.7383 (2)	0.73948 (19)	0.23512 (15)	0.0258 (5)	
O1	−0.5899 (3)	0.0147 (3)	0.1083 (2)	0.0422 (7)	
H1	−0.4899	0.0534	0.1182	0.063*	
N1	0.5942 (3)	0.6362 (3)	0.3580 (2)	0.0185 (6)	
N2	0.5944 (3)	0.4878 (3)	0.1630 (2)	0.0172 (5)	
N3	−0.2990 (3)	0.1062 (3)	0.2134 (2)	0.0273 (7)	
C1	0.6416 (4)	0.7349 (3)	0.4619 (3)	0.0216 (7)	
C2	0.5163 (4)	0.7140 (3)	0.5250 (3)	0.0248 (7)	
H2	0.5190	0.7695	0.6008	0.030*	
C3	0.3881 (4)	0.5993 (3)	0.4586 (3)	0.0214 (7)	
C4	0.4365 (4)	0.5496 (3)	0.3526 (2)	0.0177 (6)	
C5	0.3579 (4)	0.4373 (3)	0.2536 (2)	0.0176 (6)	
C6	0.4352 (4)	0.4064 (3)	0.1602 (2)	0.0178 (6)	
C7	0.3803 (4)	0.3018 (3)	0.0502 (2)	0.0191 (7)	
C8	0.5079 (4)	0.3232 (3)	−0.0097 (3)	0.0224 (7)	
H8	0.5078	0.2698	−0.0856	0.027*	
C9	0.6371 (4)	0.4374 (3)	0.0616 (3)	0.0204 (7)	
C10	0.8032 (4)	0.8467 (3)	0.4960 (3)	0.0290 (8)	
H10A	0.8851	0.8034	0.4958	0.043*	
H10B	0.8089	0.9145	0.5710	0.043*	
H10C	0.8218	0.8956	0.4434	0.043*	
C11	0.2300 (4)	0.5436 (4)	0.4972 (3)	0.0275 (8)	
H11A	0.2004	0.4414	0.4771	0.041*	
H11B	0.1473	0.5634	0.4612	0.041*	
H11C	0.2399	0.5897	0.5782	0.041*	
C12	0.2217 (4)	0.1875 (3)	0.0032 (3)	0.0261 (8)	
H12A	0.2071	0.1566	−0.0782	0.039*	
H12B	0.1364	0.2234	0.0325	0.039*	
H12C	0.2174	0.1075	0.0245	0.039*	
C13	0.8007 (4)	0.4954 (4)	0.0331 (3)	0.0260 (7)	
H13A	0.8350	0.5986	0.0611	0.039*	
H13B	0.7977	0.4587	−0.0480	0.039*	
H13C	0.8763	0.4667	0.0674	0.039*	
C14	0.1879 (4)	0.3498 (3)	0.2468 (2)	0.0186 (7)	
C15	0.0661 (4)	0.3993 (3)	0.2304 (3)	0.0214 (7)	
H15	0.0923	0.4909	0.2278	0.026*	
C16	−0.0932 (4)	0.3161 (3)	0.2177 (3)	0.0230 (7)	
H16	−0.1758	0.3485	0.2025	0.028*	
C17	−0.1317 (4)	0.1848 (3)	0.2273 (3)	0.0211 (7)	
C18	−0.0107 (4)	0.1359 (3)	0.2457 (3)	0.0240 (7)	
H18	−0.0368	0.0467	0.2527	0.029*	
C19	0.1484 (4)	0.2175 (3)	0.2539 (3)	0.0218 (7)	
H19	0.2308	0.1826	0.2645	0.026*	
C20	−0.3510 (4)	0.0379 (3)	0.2744 (3)	0.0273 (8)	
H20	−0.2780	0.0384	0.3292	0.033*	
C21	−0.5211 (4)	−0.0427 (3)	0.2635 (3)	0.0269 (8)	
C22	−0.6322 (4)	−0.0491 (3)	0.1813 (3)	0.0294 (8)	
C23	−0.7950 (4)	−0.1246 (4)	0.1738 (3)	0.0355 (9)	
H23	−0.8707	−0.1302	0.1181	0.043*	
C24	−0.8441 (4)	−0.1901 (4)	0.2471 (3)	0.0360 (9)	
H24	−0.9546	−0.2405	0.2426	0.043*	
C25	−0.7343 (5)	−0.1839 (4)	0.3286 (3)	0.0375 (9)	
H25	−0.7702	−0.2302	0.3789	0.045*	
C26	−0.5743 (5)	−0.1111 (4)	0.3362 (3)	0.0329 (9)	
H26	−0.4998	−0.1076	0.3916	0.039*	
B1	0.6956 (4)	0.6192 (4)	0.2632 (3)	0.0195 (7)	

### Atomic displacement parameters (Å^2^)

**Table:**

	*U*^11^	*U*^22^	*U*^33^	*U*^12^	*U*^13^	*U*^23^
F1	0.0179 (9)	0.0254 (10)	0.0253 (10)	0.0075 (8)	0.0021 (8)	0.0066 (8)
F2	0.0269 (10)	0.0216 (9)	0.0261 (10)	0.0038 (8)	0.0033 (8)	0.0098 (8)
O1	0.0268 (14)	0.0541 (18)	0.0488 (17)	0.0098 (14)	−0.0003 (13)	0.0267 (15)
N1	0.0176 (13)	0.0181 (12)	0.0159 (13)	0.0047 (10)	0.0004 (10)	0.0029 (10)
N2	0.0160 (13)	0.0191 (12)	0.0155 (13)	0.0063 (10)	0.0038 (10)	0.0052 (10)
N3	0.0239 (15)	0.0224 (14)	0.0331 (16)	0.0067 (12)	0.0118 (13)	0.0079 (13)
C1	0.0250 (17)	0.0182 (15)	0.0190 (16)	0.0086 (13)	0.0000 (13)	0.0027 (13)
C2	0.0324 (19)	0.0231 (16)	0.0151 (15)	0.0110 (14)	0.0040 (14)	0.0011 (13)
C3	0.0245 (17)	0.0229 (16)	0.0186 (16)	0.0117 (13)	0.0048 (13)	0.0066 (13)
C4	0.0178 (15)	0.0177 (14)	0.0171 (15)	0.0053 (12)	0.0039 (12)	0.0063 (12)
C5	0.0181 (15)	0.0160 (14)	0.0193 (15)	0.0073 (12)	0.0025 (12)	0.0059 (12)
C6	0.0167 (15)	0.0169 (14)	0.0176 (15)	0.0051 (12)	0.0001 (12)	0.0046 (12)
C7	0.0217 (16)	0.0206 (15)	0.0156 (15)	0.0104 (13)	0.0007 (12)	0.0047 (13)
C8	0.0261 (17)	0.0235 (16)	0.0164 (15)	0.0099 (14)	0.0053 (13)	0.0047 (13)
C9	0.0213 (16)	0.0251 (16)	0.0192 (16)	0.0128 (13)	0.0071 (13)	0.0087 (13)
C10	0.0288 (19)	0.0207 (16)	0.0249 (18)	0.0017 (14)	−0.0013 (14)	−0.0001 (14)
C11	0.0307 (19)	0.0297 (18)	0.0207 (17)	0.0111 (15)	0.0114 (14)	0.0066 (14)
C12	0.0249 (18)	0.0234 (16)	0.0213 (17)	0.0043 (14)	−0.0049 (14)	0.0018 (14)
C13	0.0235 (17)	0.0340 (18)	0.0242 (18)	0.0122 (15)	0.0100 (14)	0.0126 (15)
C14	0.0178 (15)	0.0194 (15)	0.0158 (15)	0.0051 (12)	0.0037 (12)	0.0042 (12)
C15	0.0202 (16)	0.0188 (15)	0.0243 (17)	0.0074 (13)	0.0072 (13)	0.0061 (13)
C16	0.0210 (16)	0.0226 (16)	0.0283 (18)	0.0093 (13)	0.0059 (14)	0.0111 (14)
C17	0.0174 (15)	0.0189 (15)	0.0221 (16)	0.0037 (12)	0.0047 (13)	0.0041 (13)
C18	0.0243 (17)	0.0184 (15)	0.0251 (17)	0.0046 (13)	0.0035 (14)	0.0056 (13)
C19	0.0203 (16)	0.0223 (15)	0.0219 (16)	0.0075 (13)	0.0020 (13)	0.0072 (13)
C20	0.0262 (18)	0.0245 (17)	0.0284 (19)	0.0117 (15)	0.0027 (15)	0.0037 (15)
C21	0.0240 (17)	0.0195 (16)	0.0313 (19)	0.0086 (14)	0.0047 (15)	0.0016 (14)
C22	0.032 (2)	0.0220 (17)	0.037 (2)	0.0103 (15)	0.0113 (16)	0.0136 (16)
C23	0.0208 (18)	0.0281 (18)	0.053 (2)	0.0094 (15)	0.0026 (17)	0.0091 (18)
C24	0.0215 (18)	0.0222 (17)	0.055 (2)	0.0030 (15)	0.0114 (18)	0.0067 (17)
C25	0.036 (2)	0.0267 (18)	0.045 (2)	0.0083 (17)	0.0147 (19)	0.0104 (17)
C26	0.038 (2)	0.0262 (18)	0.038 (2)	0.0154 (16)	0.0110 (17)	0.0119 (16)
B1	0.0170 (17)	0.0195 (16)	0.0197 (18)	0.0061 (14)	0.0024 (14)	0.0047 (14)

### Geometric parameters (Å, º)

**Table:**

F1—B1	1.397 (4)	C11—H11B	0.9800
F2—B1	1.402 (4)	C11—H11C	0.9800
O1—C22	1.341 (4)	C12—H12A	0.9800
O1—H1	0.8400	C12—H12B	0.9800
N1—C1	1.348 (4)	C12—H12C	0.9800
N1—C4	1.402 (4)	C13—H13A	0.9800
N1—B1	1.528 (4)	C13—H13B	0.9800
N2—C9	1.345 (4)	C13—H13C	0.9800
N2—C6	1.401 (4)	C14—C19	1.389 (4)
N2—B1	1.541 (4)	C14—C15	1.392 (4)
N3—C20	1.254 (4)	C15—C16	1.387 (4)
N3—C17	1.432 (4)	C15—H15	0.9500
C1—C2	1.398 (4)	C16—C17	1.393 (4)
C1—C10	1.492 (4)	C16—H16	0.9500
C2—C3	1.379 (4)	C17—C18	1.388 (5)
C2—H2	0.9500	C18—C19	1.389 (4)
C3—C4	1.416 (4)	C18—H18	0.9500
C3—C11	1.510 (4)	C19—H19	0.9500
C4—C5	1.397 (4)	C20—C21	1.462 (5)
C5—C6	1.390 (4)	C20—H20	0.9500
C5—C14	1.489 (4)	C21—C26	1.386 (5)
C6—C7	1.431 (4)	C21—C22	1.407 (5)
C7—C8	1.388 (4)	C22—C23	1.401 (5)
C7—C12	1.493 (4)	C23—C24	1.366 (5)
C8—C9	1.402 (4)	C23—H23	0.9500
C8—H8	0.9500	C24—C25	1.394 (6)
C9—C13	1.498 (4)	C24—H24	0.9500
C10—H10A	0.9800	C25—C26	1.373 (5)
C10—H10B	0.9800	C25—H25	0.9500
C10—H10C	0.9800	C26—H26	0.9500
C11—H11A	0.9800		
			
C22—O1—H1	109.5	H12B—C12—H12C	109.5
C1—N1—C4	108.1 (2)	C9—C13—H13A	109.5
C1—N1—B1	126.1 (3)	C9—C13—H13B	109.5
C4—N1—B1	125.7 (3)	H13A—C13—H13B	109.5
C9—N2—C6	108.2 (2)	C9—C13—H13C	109.5
C9—N2—B1	126.7 (3)	H13A—C13—H13C	109.5
C6—N2—B1	124.9 (2)	H13B—C13—H13C	109.5
C20—N3—C17	120.2 (3)	C19—C14—C15	119.2 (3)
N1—C1—C2	109.0 (3)	C19—C14—C5	121.0 (3)
N1—C1—C10	122.3 (3)	C15—C14—C5	119.7 (3)
C2—C1—C10	128.7 (3)	C16—C15—C14	120.6 (3)
C3—C2—C1	108.6 (3)	C16—C15—H15	119.7
C3—C2—H2	125.7	C14—C15—H15	119.7
C1—C2—H2	125.7	C15—C16—C17	119.7 (3)
C2—C3—C4	106.5 (3)	C15—C16—H16	120.1
C2—C3—C11	124.4 (3)	C17—C16—H16	120.1
C4—C3—C11	129.2 (3)	C18—C17—C16	119.9 (3)
C5—C4—N1	120.1 (3)	C18—C17—N3	124.0 (3)
C5—C4—C3	132.1 (3)	C16—C17—N3	116.1 (3)
N1—C4—C3	107.8 (3)	C17—C18—C19	120.0 (3)
C6—C5—C4	121.0 (3)	C17—C18—H18	120.0
C6—C5—C14	119.2 (3)	C19—C18—H18	120.0
C4—C5—C14	119.8 (3)	C14—C19—C18	120.5 (3)
C5—C6—N2	120.8 (3)	C14—C19—H19	119.8
C5—C6—C7	131.5 (3)	C18—C19—H19	119.8
N2—C6—C7	107.7 (2)	N3—C20—C21	121.8 (3)
C8—C7—C6	106.3 (3)	N3—C20—H20	119.1
C8—C7—C12	124.0 (3)	C21—C20—H20	119.1
C6—C7—C12	129.7 (3)	C26—C21—C22	119.5 (3)
C7—C8—C9	108.0 (3)	C26—C21—C20	119.8 (3)
C7—C8—H8	126.0	C22—C21—C20	120.7 (3)
C9—C8—H8	126.0	O1—C22—C23	117.4 (3)
N2—C9—C8	109.7 (3)	O1—C22—C21	122.9 (3)
N2—C9—C13	123.8 (3)	C23—C22—C21	119.7 (3)
C8—C9—C13	126.5 (3)	C24—C23—C22	119.5 (4)
C1—C10—H10A	109.5	C24—C23—H23	120.3
C1—C10—H10B	109.5	C22—C23—H23	120.3
H10A—C10—H10B	109.5	C23—C24—C25	120.9 (3)
C1—C10—H10C	109.5	C23—C24—H24	119.5
H10A—C10—H10C	109.5	C25—C24—H24	119.5
H10B—C10—H10C	109.5	C26—C25—C24	120.1 (4)
C3—C11—H11A	109.5	C26—C25—H25	120.0
C3—C11—H11B	109.5	C24—C25—H25	120.0
H11A—C11—H11B	109.5	C25—C26—C21	120.3 (4)
C3—C11—H11C	109.5	C25—C26—H26	119.9
H11A—C11—H11C	109.5	C21—C26—H26	119.9
H11B—C11—H11C	109.5	F1—B1—F2	108.0 (3)
C7—C12—H12A	109.5	F1—B1—N1	110.6 (3)
C7—C12—H12B	109.5	F2—B1—N1	110.3 (3)
H12A—C12—H12B	109.5	F1—B1—N2	110.0 (3)
C7—C12—H12C	109.5	F2—B1—N2	110.5 (3)
H12A—C12—H12C	109.5	N1—B1—N2	107.4 (2)
			
C4—N1—C1—C2	−0.1 (4)	C4—C5—C14—C19	−102.0 (4)
B1—N1—C1—C2	−178.8 (3)	C6—C5—C14—C15	−99.7 (4)
C4—N1—C1—C10	−178.7 (3)	C4—C5—C14—C15	79.6 (4)
B1—N1—C1—C10	2.6 (5)	C19—C14—C15—C16	−2.0 (5)
N1—C1—C2—C3	0.2 (4)	C5—C14—C15—C16	176.4 (3)
C10—C1—C2—C3	178.7 (3)	C14—C15—C16—C17	3.4 (5)
C1—C2—C3—C4	−0.2 (4)	C15—C16—C17—C18	−2.1 (5)
C1—C2—C3—C11	180.0 (3)	C15—C16—C17—N3	179.7 (3)
C1—N1—C4—C5	−179.7 (3)	C20—N3—C17—C18	41.8 (4)
B1—N1—C4—C5	−1.0 (5)	C20—N3—C17—C16	−140.2 (3)
C1—N1—C4—C3	−0.1 (3)	C16—C17—C18—C19	−0.4 (5)
B1—N1—C4—C3	178.6 (3)	N3—C17—C18—C19	177.6 (3)
C2—C3—C4—C5	179.7 (3)	C15—C14—C19—C18	−0.6 (5)
C11—C3—C4—C5	−0.4 (6)	C5—C14—C19—C18	−179.0 (3)
C2—C3—C4—N1	0.2 (3)	C17—C18—C19—C14	1.8 (5)
C11—C3—C4—N1	180.0 (3)	C17—N3—C20—C21	179.5 (3)
N1—C4—C5—C6	2.1 (5)	N3—C20—C21—C26	−176.9 (3)
C3—C4—C5—C6	−177.4 (3)	N3—C20—C21—C22	1.7 (5)
N1—C4—C5—C14	−177.2 (3)	C26—C21—C22—O1	179.8 (3)
C3—C4—C5—C14	3.3 (5)	C20—C21—C22—O1	1.1 (5)
C4—C5—C6—N2	−0.2 (5)	C26—C21—C22—C23	−0.1 (5)
C14—C5—C6—N2	179.0 (3)	C20—C21—C22—C23	−178.7 (3)
C4—C5—C6—C7	−177.9 (3)	O1—C22—C23—C24	−179.3 (3)
C14—C5—C6—C7	1.4 (5)	C21—C22—C23—C24	0.6 (5)
C9—N2—C6—C5	−178.4 (3)	C22—C23—C24—C25	−0.6 (5)
B1—N2—C6—C5	−2.9 (4)	C23—C24—C25—C26	0.2 (5)
C9—N2—C6—C7	−0.3 (3)	C24—C25—C26—C21	0.4 (5)
B1—N2—C6—C7	175.3 (3)	C22—C21—C26—C25	−0.4 (5)
C5—C6—C7—C8	178.0 (3)	C20—C21—C26—C25	178.3 (3)
N2—C6—C7—C8	0.1 (3)	C1—N1—B1—F1	56.8 (4)
C5—C6—C7—C12	−3.2 (6)	C4—N1—B1—F1	−121.7 (3)
N2—C6—C7—C12	179.0 (3)	C1—N1—B1—F2	−62.7 (4)
C6—C7—C8—C9	0.1 (4)	C4—N1—B1—F2	118.8 (3)
C12—C7—C8—C9	−178.9 (3)	C1—N1—B1—N2	176.9 (3)
C6—N2—C9—C8	0.3 (3)	C4—N1—B1—N2	−1.6 (4)
B1—N2—C9—C8	−175.1 (3)	C9—N2—B1—F1	−61.3 (4)
C6—N2—C9—C13	−178.5 (3)	C6—N2—B1—F1	124.0 (3)
B1—N2—C9—C13	6.1 (5)	C9—N2—B1—F2	57.9 (4)
C7—C8—C9—N2	−0.2 (4)	C6—N2—B1—F2	−116.8 (3)
C7—C8—C9—C13	178.5 (3)	C9—N2—B1—N1	178.2 (3)
C6—C5—C14—C19	78.7 (4)	C6—N2—B1—N1	3.5 (4)

### Hydrogen-bond geometry (Å, º)

**Table:**

*D*—H···*A*	*D*—H	H···*A*	*D*···*A*	*D*—H···*A*
O1—H1···N3	0.84	1.89	2.618 (4)	145
C16—H16···F1^i^	0.95	2.53	3.150 (5)	123
C24—H24···F1^ii^	0.95	2.37	3.235 (5)	151

Symmetry codes: (i) *x*−1, *y*, *z*; (ii) *x*−2, *y*−1, *z*.
